# The Funeral of Dr. Thomas W. Evans

**Published:** 1898-07

**Authors:** 


### THE FUNERAL OF DR. THOMAS W. EVANS.

The final ceremonies over the body of Dr. Evans took place on Wednesday, May 25, at the Protestant Episcopal Church of St. Mary, Thirty-ninth and Locust Streets, Philadelphia. The services were confined to the usual ritual of that religious denomination.

The body was placed in a lead coffin, covered with aluminum, giving the unique appearance of being encased in silver. This prevented the use of handles, and the great weight required the services of eight men to carry it upon their shoulders.

The most impressive lesson conveyed by these obsequies was the evidence given that the honors conferred upon the majority of men, unless based upon an enduring foundation, die at the entrance to the tomb. Dr. Evans was the professional adviser of emperors, kings, and nobles, and a friend of many. Yet, outside of the immediate relatives and officials, he had scarcely sixty persons to honor his remains, and the members of the dental profession, of his native city, were conspicuous by their absence. It was, to the writer, a sad reflection of the instability of all earthly honors, even though accompanied by great wealth.

Dr. Evans's real work in the dental profession has received due credit elsewhere, but it is worthy of repetition here that the labor of his life in certain directions did much to place dentistry upon a higher level of appreciation; more, perhaps, than was accomplished by any other man of his day and generation.

The body was temporarily entombed at Woodland Cemetery, in a vault specially prepared, in which his wife had previously been laid. Here they will remain until the mausoleum is built, provided for in his will. When this may be it is difficult to say, for while the will has been probated, the contending heirs seem determined to carry it through the courts, and it may be months or years before litigation is ended. When it is finally sustained, as doubtless it will be, there will be other difficulties to be met in the establishment of his college and museum, the fulfilment of his aspirations and an evidence of his devotion to his profession.

